# Supplementary material for: Celiac-Related Autoantibodies and IL-17A in Bulgarian Patients with Dermatitis Herpetiformis: A Cross-Sectional Study
Source: Medicina (Kaunas). 2019 May 15;55(5):136. doi: 10.3390/medicina55050136 (PMC6572648; doi:10.3390/medicina55050136)
Supplement: Supplementary file 1 [file medicina-55-00136-s001.pdf]

Table 1. Serum levels of the celiac-related antibodies and IL-17A in DH patients and controls, investigated by ELISA and immunoblot. Results are presented as Mean ± SE (Range).

		Dermatitis Herpetiformis Patients	Healthy Individuals	P Value*
ELISA	Anti-tTG, U/ml	36.9 ± 20.3 (1.5 – 200)	2.1 ± 0.4 (0 – 3.1)	0.02
	Anti-DGP, U/ml	40.7 ± 10.2 (2.8 – 163)	1.87 ± 0.68 (0 – 2.5)	<0.001
	AAA, U/ml	22.6 ± 3.9 (4.6 – 90.5)	9.1 ± 0.9 (0 – 10)	0.05
	IL-17A, pg/ml	5.3 ± 2.2 (0 – 46.9)	0.08 ± 0.07 (0 – 1.1)	0.031
Blot	Anti-tTG**	0.88 ± 0.24 (0 - 4.0)	0.08 ± 0.02 (0 - 0.2)	0.003
	AGA**	0.98 ± 0.31 (0 – 5.2)	0.25 ± 0.08 (0 - 1.1)	0.030
	ASCA**	0.028 ± 0.1 (0 - 0.2)	0.04 ± 0.02 (0 - 0.2)	>0.05

*Differences between both study groups are calculated by Independent T-test.

**Results are presented as the relative value of line blot intensity

Table 2. Receiver operating characteristics (ROC) curve analysis of the tested parameters assessed by ELISA and Line blot.

	Parameter	Area under the curve (AUC)	95% CI	P value
ELISA	Anti-tTG antibodies	0.864	0.724 – 1.000	0.002
	Anti-DGP antibodies	0.939	0.856 – 1.000	<0.001
	AAA	0.689	0.451 – 0.927	>0.05
	IL-17A	0.811	0. 608 – 1.000	0.008
Blot	Anti-tTG antibodies	0.734	0.587 – 0.881	0.002
	AGA	0.600	0.432 – 0.768	>0.05
	ASCA	0.452	0.280 – 0.624	>0.05
